# Supplementary material for: A multi-modal, asymmetric, weighted, and signed description of anatomical connectivity
Source: Nat Commun. 2024 Jul 12;15:5865. doi: 10.1038/s41467-024-50248-6 (PMC11245624; doi:10.1038/s41467-024-50248-6)
Supplement: Supplementary file 1 — Supplementary information [file 41467_2024_50248_MOESM1_ESM.pdf]

SUPPLEMENTARY MATERIALS

In this supplemental section, we describe the results of analyses that support the results and arguments made in our main text.

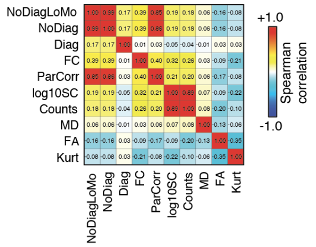

FIG. S1. **Similarity of edge weights.** In the main text we use linear models to fit edge weights to structural connections. Here, we different edge weighting schemes. We consider: the logarithm of fiber densities (log10SC), estimated edge weights using all data (NoDiag), estimated edge weights using only low-motion data (NoDiagLoMo), estimated edge weights with the inclusion of an autoregressive diagonal term (Diag), functional connectivity estimated using a full correlation (FC), and partial correlations (ParCorr). In all cases, similarity was calculated as the Spearman correlation using only edges for which a structural connection was observed.

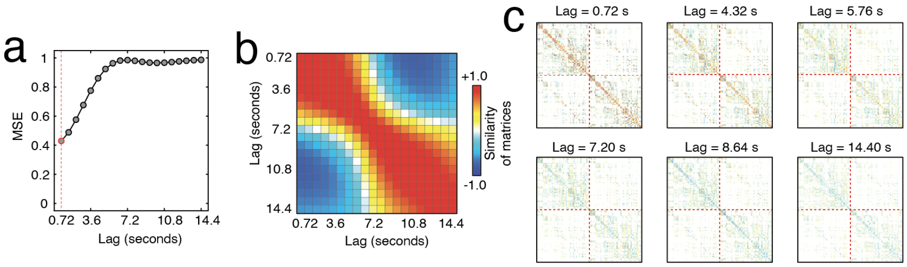

FIG. S2. **Effect of lag on estimated edge weights.** In the main text we use linear models to fit edge weights by predicting activity at time $t+1$ using activity at time t (one frame of lag = 0.720 seconds). Here, we investigate the effect of longer lags on the estimated weights, increasing the lag to ≈15 seconds. (a) Model error at different lags. (b) Spatial similarity of weights estimated at each lab. (c) Example weight matrices at different lags.

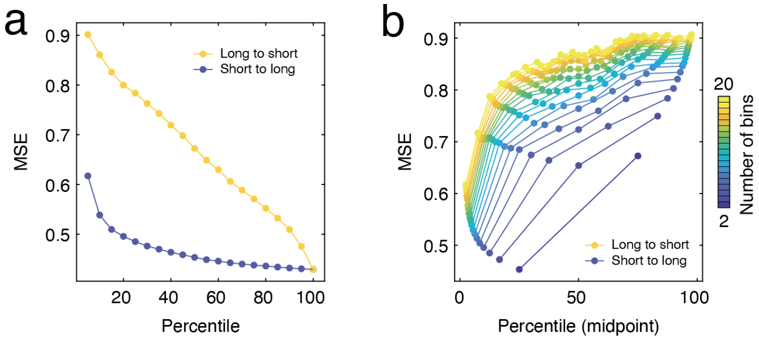

FIG. S3. **Effects of connection length on model performance.** We divided connections into 20 percentile bins based on their length. Next, we created structural network backbone comprising the 5% shortest (or longest) connections, gradually adding back the progressively longer (or shorter) connection 19 bins, until all connections were added. This allowed us to assess the relative contributions of short *versus* long connections. In panel a, we plot the model error as a function across all percentiles. We also grouped connections by percentiles (from 2 to 20) and fit the model using each percentile independently rather cumulatively. The results in panel b suggest that short connections are more informative and lead to reduced model error.

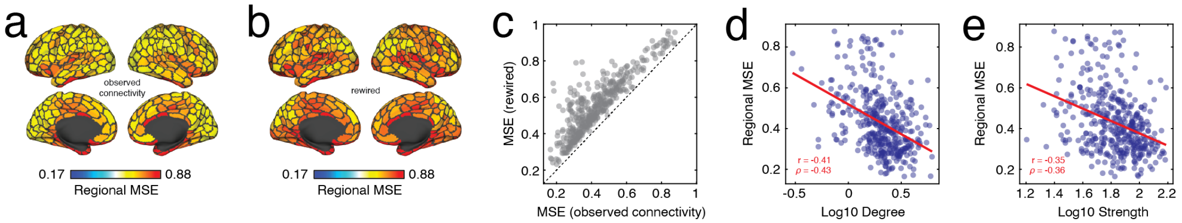

FIG. S4. **Model errors at the regional level.** Regional mean squared error (MSE) for model fit using observed and intact network (*a*) *versus* the mean MSE across 100 models fit using a degree-preserving rewiring model (*b*). (*c*) Scatterplot comparing regional error. Points above the diagonal are regions fit better using the observed network compared to the rewired. Panels *d* and *e* compare regional MSE with the logarithm of nodes' degree and weighted degree. That is, nodes with fewer connections exhibited worse fits (greater MSE).

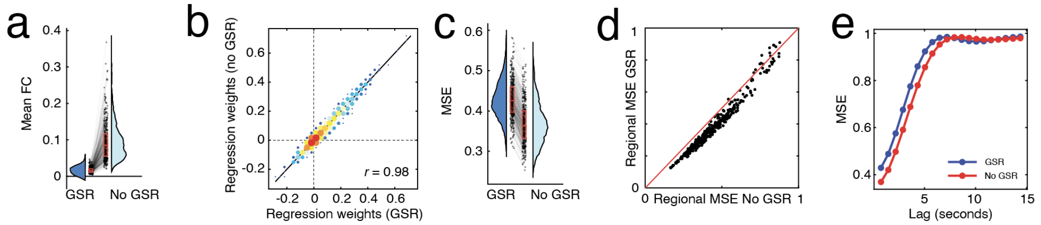

FIG. S5. **Effect of global signal regression on results.** The human data analyzed in the main text was processed using a procedure that included global signal regression (GSR). Here, we compare select results using data processed without GSR. (*a*) Mean FC for GSR and non-GSR data; each point is a scan (4 scans $\times$ 95 participants yields 380 points). (*b*) Two-dimensional histogram of group-level edge weights estimated using GSR and non-GSR data. (*c*) Subject-level model error ($N = 95$). (*d*) Comparison of regional model errors. The red line is identity (equal performance with both models). Points below the line have time series better predicted with non-GSR data; points above the line have activity time series better predicted by GSR data. (*e*) Model error as a function of lag.

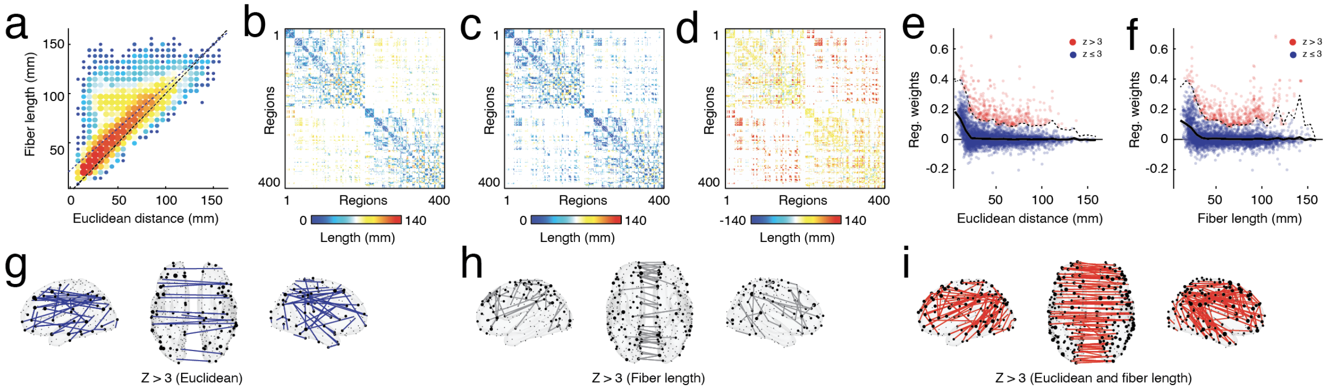

FIG. S6. **Dependence of distance effects on Euclidean distance *versus* fiber length.** (*a*) Euclidean distance *versus* fiber length. Panels *b* and *c* depict edges weighted by fiber fiber length and Euclidean distance. (*d*) Difference in fiber length and Euclidean distance. Panels *e* and *f* show connection weight versus distance. Panels *g*-*i* show connections that are stronger than expected but unique to Euclidean distance, fiber length, or are shared between both.

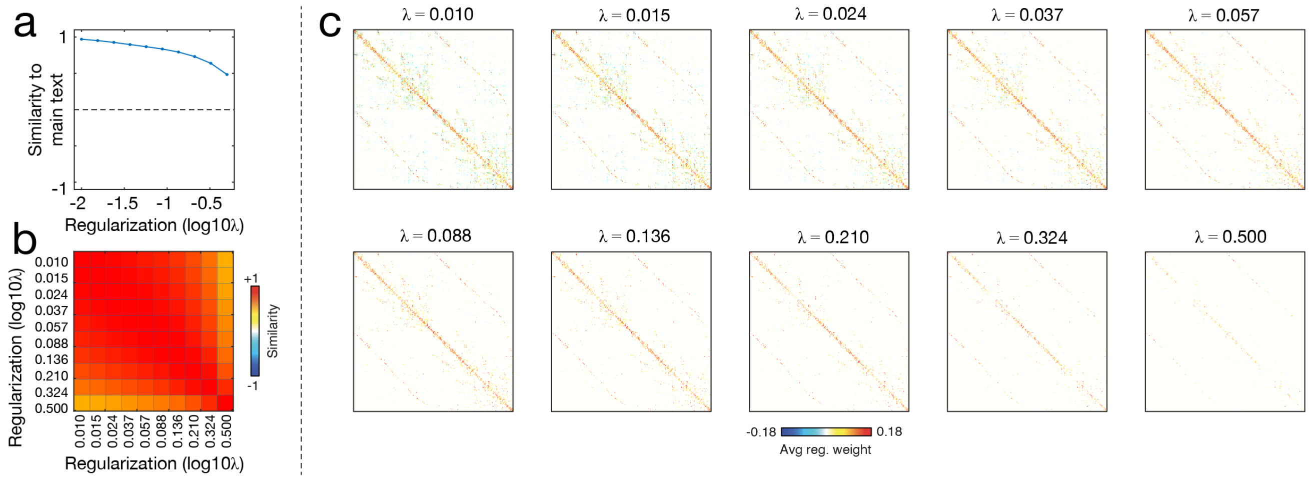

FIG. S7. **Comparing connectivity weights estimated with and without regularization.** In the main text we used linear regression optimized with ordinary least squares to fit weights to edges. Here, we compare those results to weights fit using lasso regularization (implemented in MATLAB with the function `lasso.m`). For each node, we fits its edge weights independently. We logarithmically sampled 10 values of the regularization parameter, λ, over the interval $[10^{-3}, 0.5]$. For each value of λ, we generate a new estimate of connection weights, with sparsity increasing monotonically. (*a*) Correlation of edge weight values with the edge weights reported in the main text (no regularization). (*b*) Similarity of regularized connection weights to one another. (*c*) Connectivity matrices.

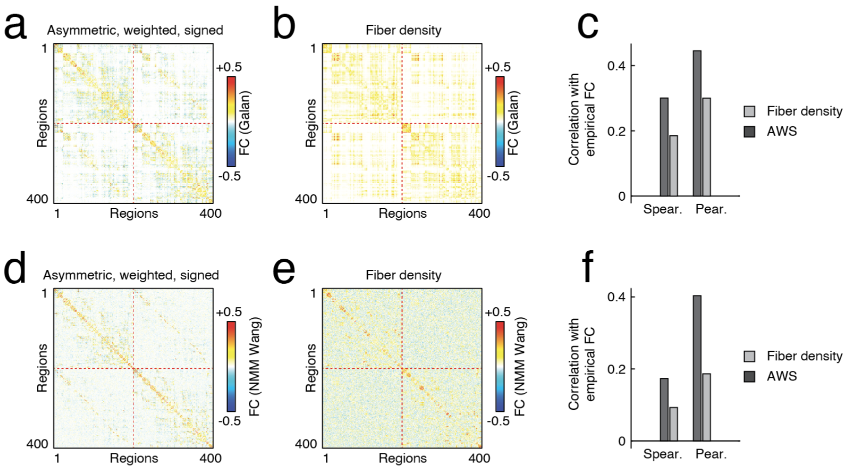

FIG. S8. **Simulated FC using linearized neural mass model.** We used a linearization of a neural mass model [1] to generate simulated covariance matrices (which are then scaled to correlation matrices; FC). We performed this procedure using both the asymmetric, weighted, and signed version of SC as well as the traditional fiber density matrix. Panels *a* and *b* show the resulting correlation matrices. We then compared these matrices to an empirical estimate of FC. We found that the correlation matrix generated using asymmetric, weighted, and signed matrix was more similar to FC than the matrix generated using the fiber density weights. Panel *c* shows the results of this analysis using both product-moment correlation and rank correlations to assess similarity. We also analyzed a simplified neural mass model (reduced Wong-Wang model with implementation made available from [2]). This procedure generates synthetic fMRI BOLD time series that can be treated identically to an empirical time series. The correlation structure of the synthetic data can then be compared to the empirical correlation structure, i.e. FC. Panels *d*-*f* depict correlation matrices for asymmetric, weighted, and signed structural connectivity, the fiber density matrix, and their correlations with respect to the empirical FC matrix.

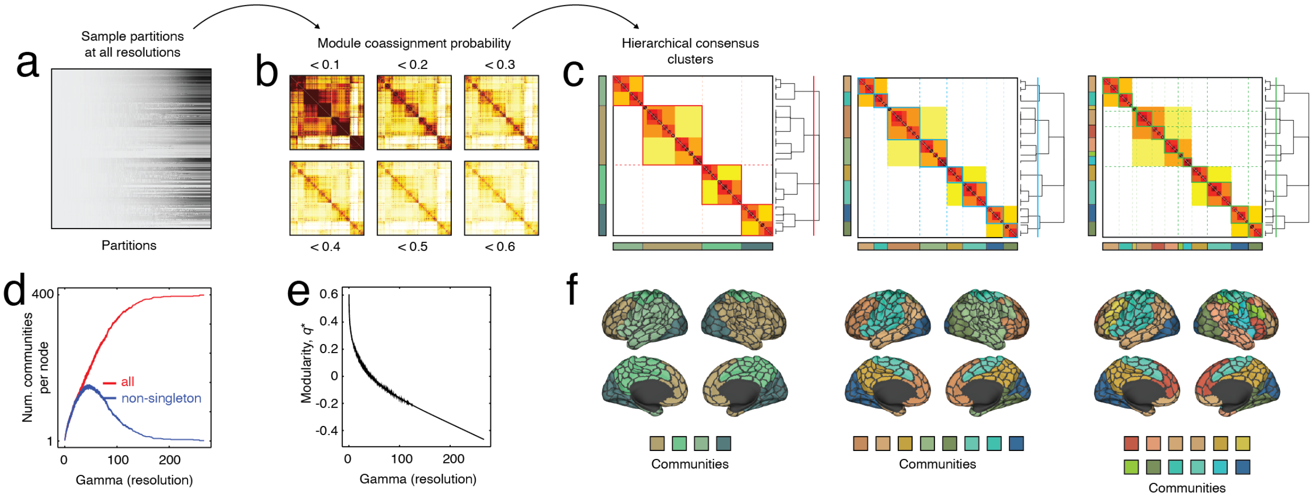

FIG. S9. **Hierarchical and multi-scale modular structure of the asymmetric, weighted, and signed connectome.** In the main text we analyzed modules at a single resolution ($\gamma = 1$). However, many networks have nested, multi-scale, and hierarchical modular organization, e.g. modules within modules within modules. To characterize this type of structure the asymmetric, weighted, and signed network, we used the "multi-resolution consensus clustering" algorithm [3]. Briefly, the algorithm works by sampling communities at many values of γ, aggregates them using a consensus clustering procedure [4], and evaluates them statistically to prune branches of the hierarchical dendrogram. (*a*) Here, we sampled 100000 values of γ at all resolutions, from divisions of the network into two communities to divisions of the network in $N-1$ communities. (*b*) For visualization, we show the module co-assignment matrix estimated using all sampled partitions. The upper range of the colormap varies to highlight different scales. The hierarchical algorithm returns a complete dendrogram (see right side of each sub-panel in *c*). In *c*, we highlight three specific resolutions, but note that there are many others. (*d*) Number of communities as a function of the resolution parameter (red) and number of non-singleton communities (blue). (*e*) Modularity of partitions. (*f*) Consensus communities for the same three hierarchical levels in *c* projected onto the cortical surface.

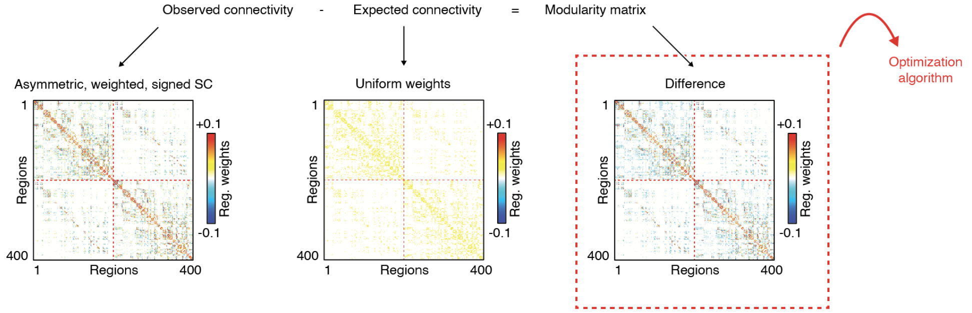

FIG. S10. **Modularity maximization with alternative, "geographic" null model.** In the main text, we described the results of detecting modules using modularity maximization with an "standard" internal null model that preserved nodes' (signed) degrees. Here, we report results using an alternative null model. Briefly, this null model preserves the same binary "backbone" of the original network, but assigns an expected weight to each connection equal to the mean weight of all existing connections. Modules therefore reflect groups of brain regions whose observed connections' weights are greater than the mean weight.

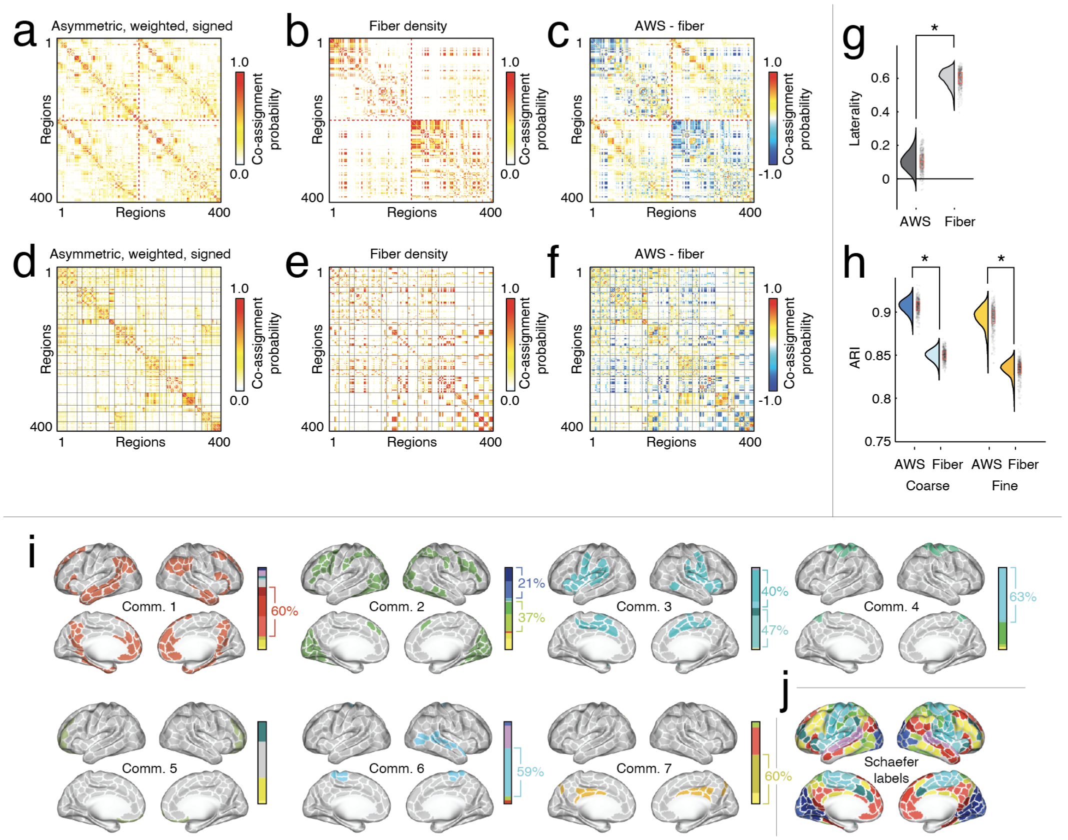

FIG. S11. **Modularity results using alternative null model.** Module coassignment matrices for the signed, weighted, and directed SC (*a*) and fiber density (*b*). (*c*) Difference in coassignment probability. Panels *d*-*f* show the same matrices, but ordered by brain system. (*g*) Laterality of detected communities. Bilateral communities have values close to zero ($N = 1000$ per box plot). (*h*) Adjusted Rand index of detected partitions with respect to coarse and fine-scale system labels ($N = 1000$ per box plot). (*i*) Consensus communities detected in signed, weighted, and directed SC. The panel next to each surface plot depicts each community's composition in terms of canonical brain systems. (*j*) Canonical brain systems.

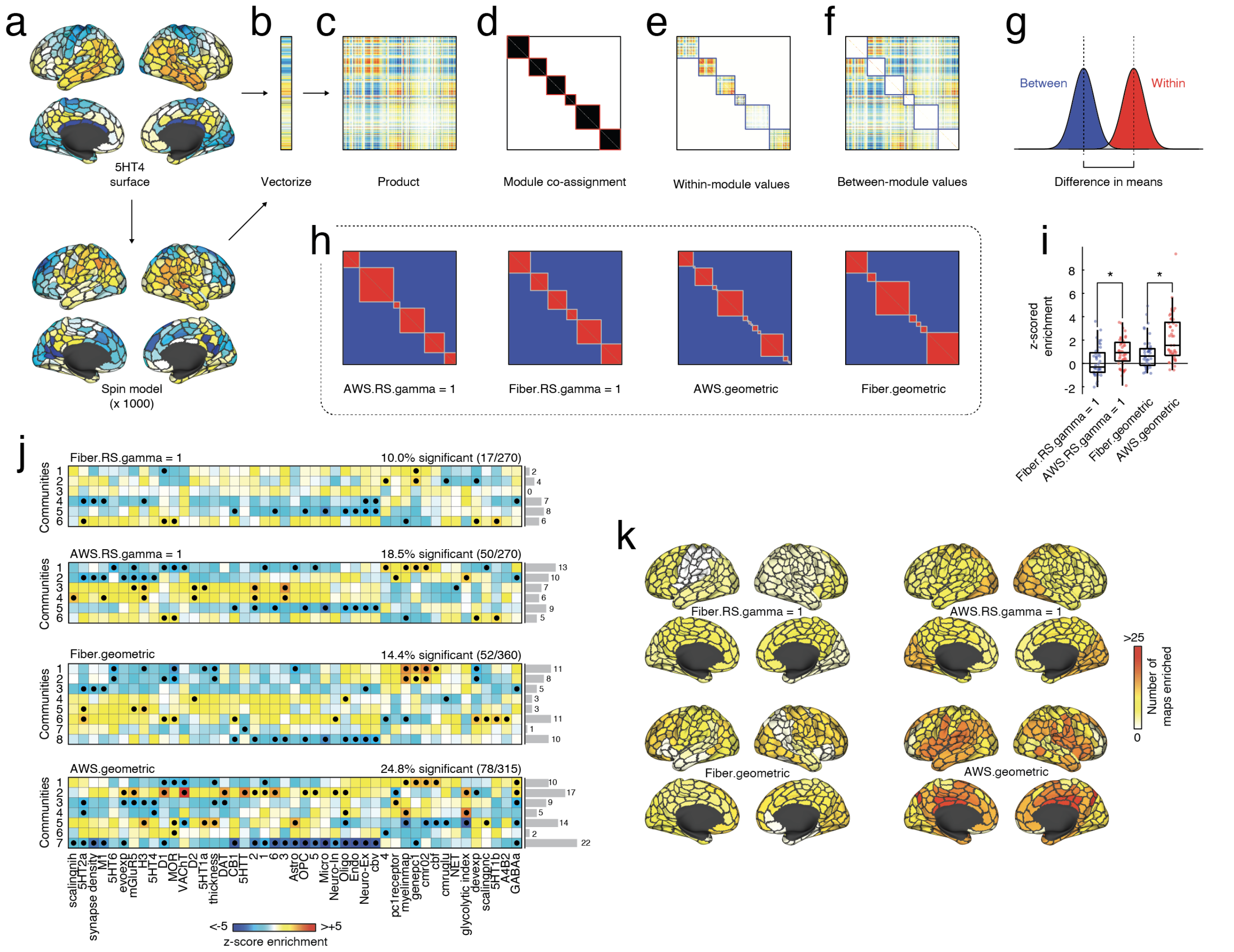

FIG. S12. **Enriching modules using brain maps.** In the main text we performed community detection on the asymmetric, weighted, and signed network (`AWS`) and fiber density network (`Fiber`) using two versions of modularity maximization: one that uses a signed internal null model (`RS`; [5])and another that we termed a "geometric" null model, in which topology is preserved but weights are not (`geometric`; [6]). Here, we further explore, validate, and compare the modules by aligning them with respect to whole-brain maps made available as part of [7]. In total, we considered 45 maps that included receptor densities, gene expression, myelination status, evolutionary and developmental expansion, and synaptic density (among others). Here, we assess whether modules are "enriched" for these maps. That is, whether the module boundaries delineate spatial boundaries in each map. The procedure for doing so is illustrated using the serotonin receptor 5HTA as an example (see panel *a* for spatial distribution on cortical surface). This map vectorized (*b*), and transformed into a matrix by calculating its outer product (*c*). We then compare the matrix to module co-assignment matrices (example(s) shown in *d* and *h*). Specifically, we calculate the mean values of all within- and between-module elements (*e* and *f*) and subsequently calculate the difference in these means (*g*). In parallel, we repeat this procedure for 1000 spatially constrained permutations of the map (spin test). The spin tests generate a null distribution against which we compare the empirical (observed) difference in means via the z-score. Larger z values indicate greater modular "enrichment". We perform this entire procedure for four sets of modules – signed and geometric model for both the asymmetric, weighted, and signed matrix as well as the fiber density matrix. Panel *i* shows the distribution of z-scores. In general, we find that the AWS model always outperforms the fiber density matrix–i.e. leads to greater z scores (paired sample t-test; maximum $p = 0.0017$). We also tested enrichment at the level of individual models (*j*). Briefly, the mean within-module value for each brain map vector was calculated and compared to a null distribution (spin tests after excluding modules of fewer than ten nodes). The z values were transformed into p values and $p < 0.05$ applied to determine statistical significance. In the margins of *j*, we show the number of maps that were significantly enriched for each module. (*k*) Number of maps enriched plotted on the cortical surface for each network/null model.

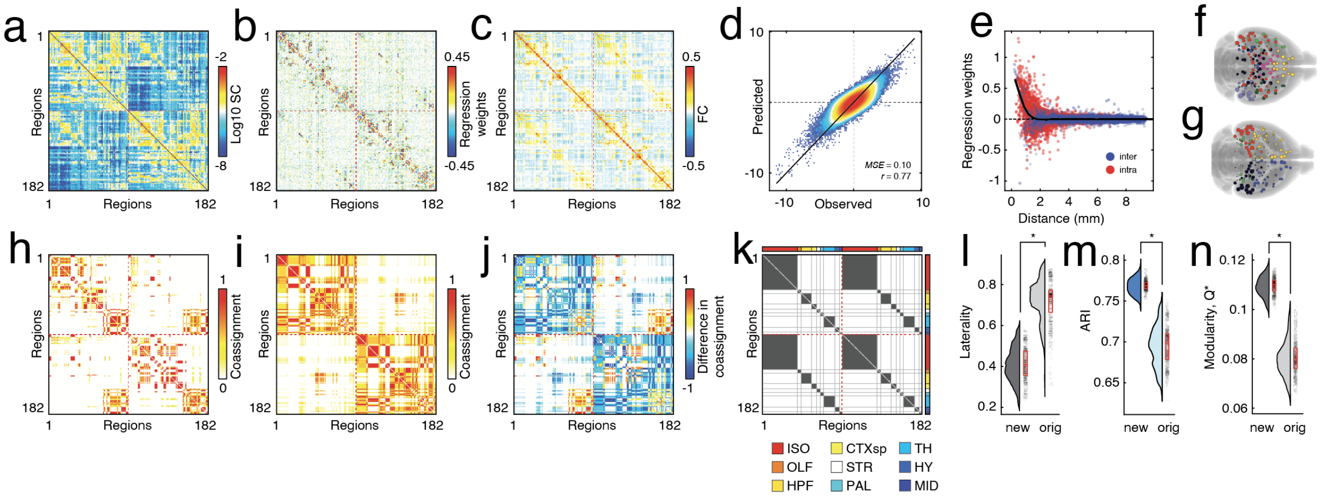

FIG. S13. **Replication of main effects using hyper-dense tract-tracing data from mice.** (a) Logarithm of anatomical connection weights. (b) Estimated weights using linear model. (c) Functional connectivity (correlation). (d) Two-dimensional histogram of predicted and observed activity across all regions and mice. (e) Scatterplot of estimated edge weights versus Euclidean distance. (f) Communities estimated by applying modularity maximization to the estimated edge weight matrix. (g) Communities estimated by applying modularity maximization to the anatomical connectivity matrix. Panels h and i show module coassignment matrices for the two versions of edge weights. Panel j shows the difference in edge weights. (k) Areal coassignment matrix. Panels l-n show laterality of detected communities, similarity of detected partitions with respect to areal labels (adjusted Rand index), and the modularity of the detected partitions ($N = 1000$ per box plot).

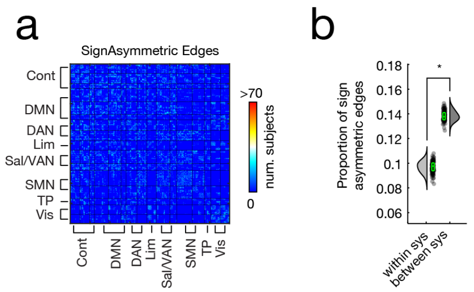

FIG. S14. **Signed asymmetry between incoming and outgoing edges is most common between functional brain systems.** (a) The number of sign asymmetric edges across 95 subjects organized by system. (b) Proportion of existing edges that exhibit sign asymmetry within systems ($N = 4654$) *versus* between systems ($N = 24370$).

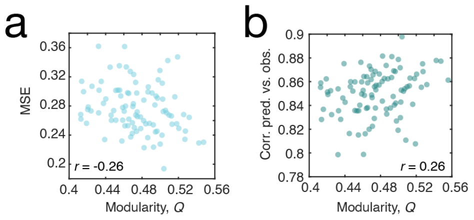

FIG. S15. **Relationship between fitness of the linear model and modularity of the functional connnectivity matrix.** (a) Relationship between modularity (Q) and mean square error (MSE). (b) Relationship between modularity (Q) and the correlation between predicted and observed time series.

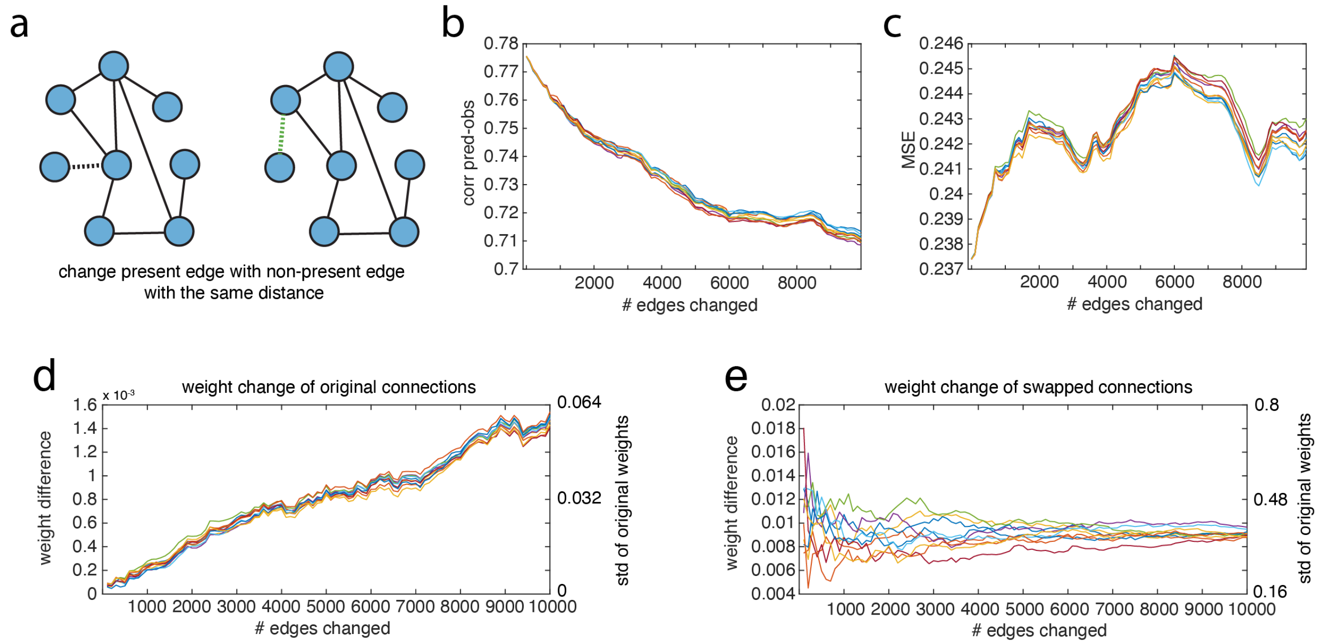

FIG. S16. **Replacing inferred SC connections with non-existent edges of the same distance degrades model performance.** (a) Schematic illustrating procedure for perturbing empirical connectome. Briefly, we identified an existing connection (shown in black) and removed it; replacing it with a new connection (shown in green) of approximately the same distance. (b) As we continued to increase the number of edges that we changed, the model performance degrades, as measured by the correlation between the predicted and observed time series. Here, we show results from 10 different runs (different colored lines). (c) As we continued to increase the number of edges that we changed, the model performance degrades (as measured by the mean squared error between the predicted and observed time series). Shown across 10 different iterations. (d) Plot showing that the weight differences remain very small between original SC edges in the intact model versus the models that contain swapped edges.(e) Plot showing that the weight differences between swapped distance-matched SC edges remain very small. Shown across 10 different iterations where swapped edges were randomly selected. Y axis represents the mean weight difference across all swapped edges. It is important to note that all of these changes were below 1 standard deviation of the weights for the model used in the main text.

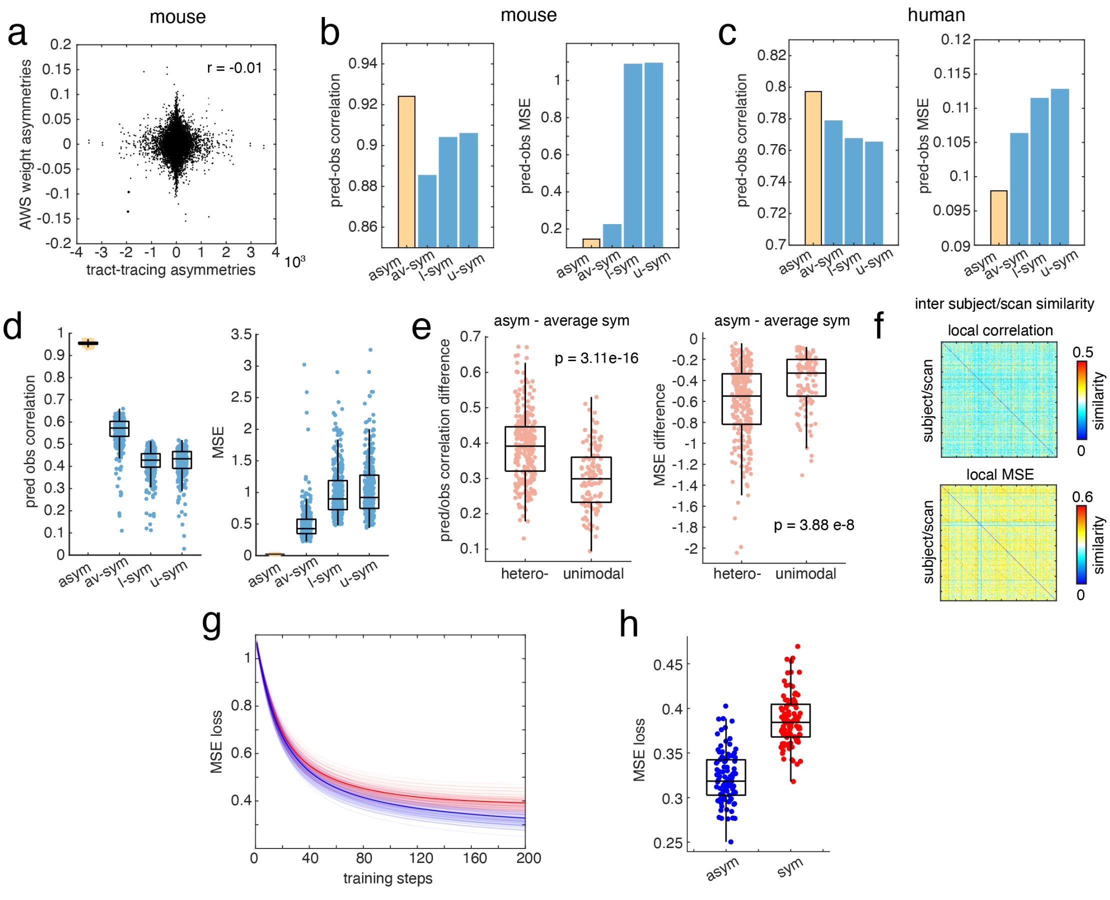

FIG. S17. **Forcing symmetry of weights degrades model performance.** (a) Scatterplot of asymmetries in weights inferred using the model proposed here with asymmetries in weights estimated directly from tract-tracing data in mouse connectome (asymmetry is measured as $W_{ij} - W_{ji}$). We explored the effect of (a)symmetry by fitting models in which we forced edge weights to be symmetric. We considered three symmetrizing routines given a pair of edges W_{ij} and W_{ji}. First, we set both edge weights equal to their mean: $W_{ji} = W_{ij} = \frac{W_{ij}+W_{ji}}{2}$. We denote the matrix estimated from this procedure as *av-sym*. The second and third approaches involved mirroring the upper and lower elements of the connectivity matrix. That is, we set both weights equal to either W_{ij} or W_{ji}. We refer to the corresponding matrices as *u-sym* and *l-sym*. We then evaluated model performance using these weights. In general, we found that model performance was best using the original asymmetric weights in both mouse (b) and human data (c). This is not surprising, as those weights were estimated to optimize model fit. (d) Next, we tracked model performance across subjects in the human data using individual level SC and similarly found that model performance is best when the original asymmetric weights are used for prediction (N = 95 per box plot). (e) Finally, we tracked model performance locally for each brain region and we found that brain regions in heteromodal systems ($N = 283$) were more negatively effected by forcing symmetry in model weights than brain regions in unimodal systems (visual and motor systems; $N = 117$). In addition, in order to check that these local changes to model performance were consistent we tracked these measures across subjects and we found that per region model performance was similar across subjects (f). The previous analyses showed that three procedures for symmetrizing weights lead to decrements in performance. It is not surprising that this is the case, as the symmetrized weights were never fit to data, making the comparison against the asymmetric weights inappropriate. As a final analysis, we use gradient descent to train regression models that estimate new weights on edges but can either allow for asymmetric weights or force weights to be symmetric. We fit these models to the 95 participants from the Human Connectome Project dataset. (g) This plot shows the loss trajectory across training for enforced symmetry (red) and models where asymmetry was allowed (blue). In general, we find that symmetric edges lead to reduced performance. Mean trajectories are colored in bold. (h) Box plots comparing the MSE loss of asymmetric (blue) and symmetric models (red) after training for 200 steps of gradient descent ($N = 95$ subjects). Asymmetric models had significantly lower loss (paired-sample t-test; $p < 10^{-15}$).

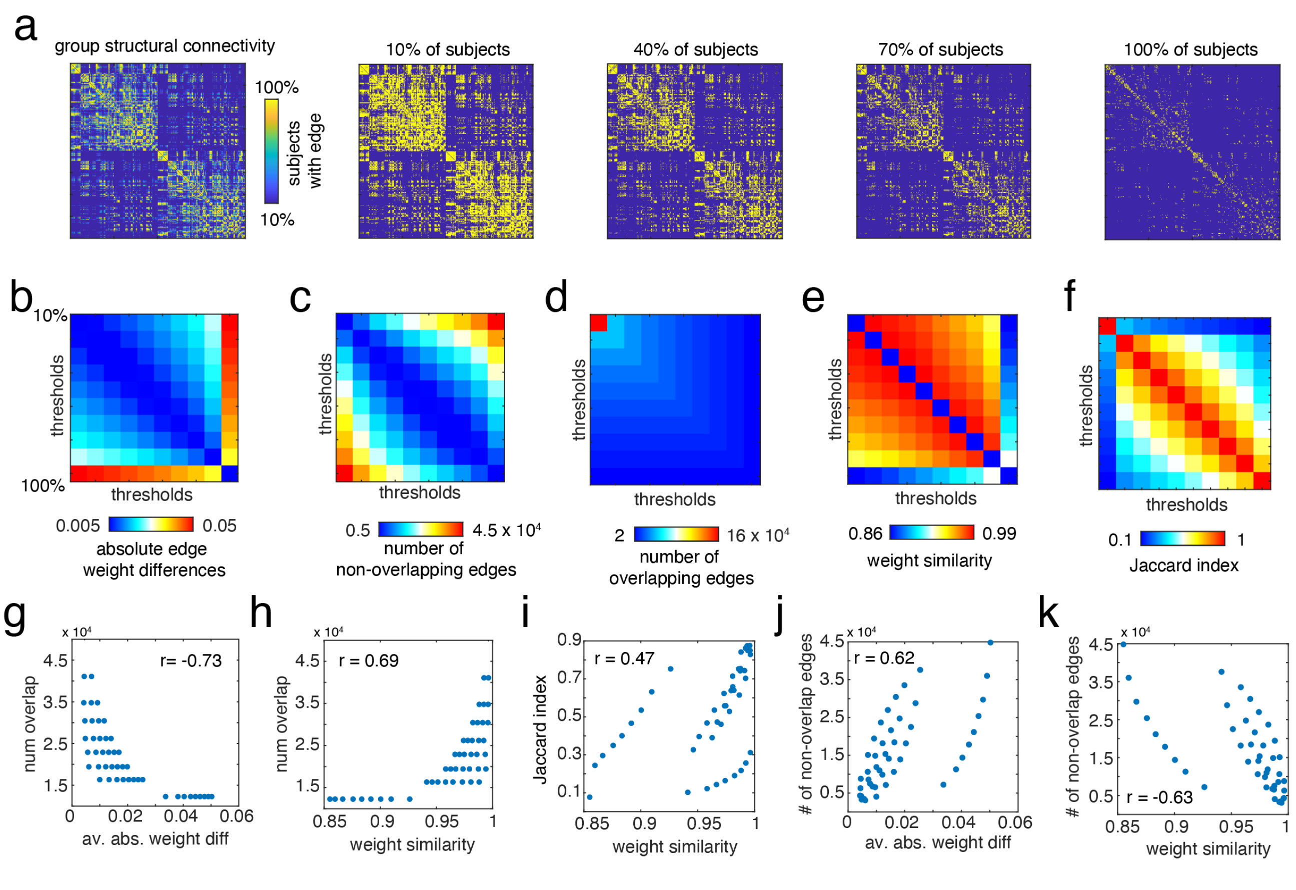

FIG. S18. **Variation in consistency-based group SC calculation: similar networks produce similar weights.** (*a*) Group structural connectivity matrices. The first matrix is a consistency matrix that shows the percentage of subjects across whom different structural connections are consistently present. The next set of matrices show how you can form different group-based structural connectivity matrices by thresholding the consistency matrix. We ran our model on the structural matrices produced across ten thresholds (10%, 20%, 30%, 40%, 50%, 60%, 70%, 80%, 90%, 100%). In the following analyses, we compare the asymmetric, and signed weights of our model across these structural connectomes. More specifically, we compare the weights of edges that are present across all thresholds. (*b*) Matrix of the absolute edge weight differences between each of these thresholded models. (*c*) Matrix of the number of non-overlapping edges between each of these thresholded SC matrices. (*d*) Matrix of the number of overlapping edges between each of these thresholded SC matrices. (*e*) Matrix of the Pearson correlation of weights between each of these thresholded models. (*f*) Matrix of the Jaccard similarity between each of these thresholded SC matrices. (*g*) Plot showing the negative relationship between the average absolute weight difference of edges and the number of overlapping edges in the thresholded SC. (*h*) Plot showing the positive relationship between the weight similarity of edges and the number of overlapping edges in the thresholded SC. (*i*) Plot showing the positive relationship between the weight similarity of edges and the Jaccard similarity of the thresholded SC. (*j*) Plot showing the positive relationship between the average absolute weight difference of edges and the number of overlapping edges in the thresholded SC. (*k*) Plot showing the negative relationship between the weight similarity of edges and the number of non-overlapping edges in the thresholded SC.

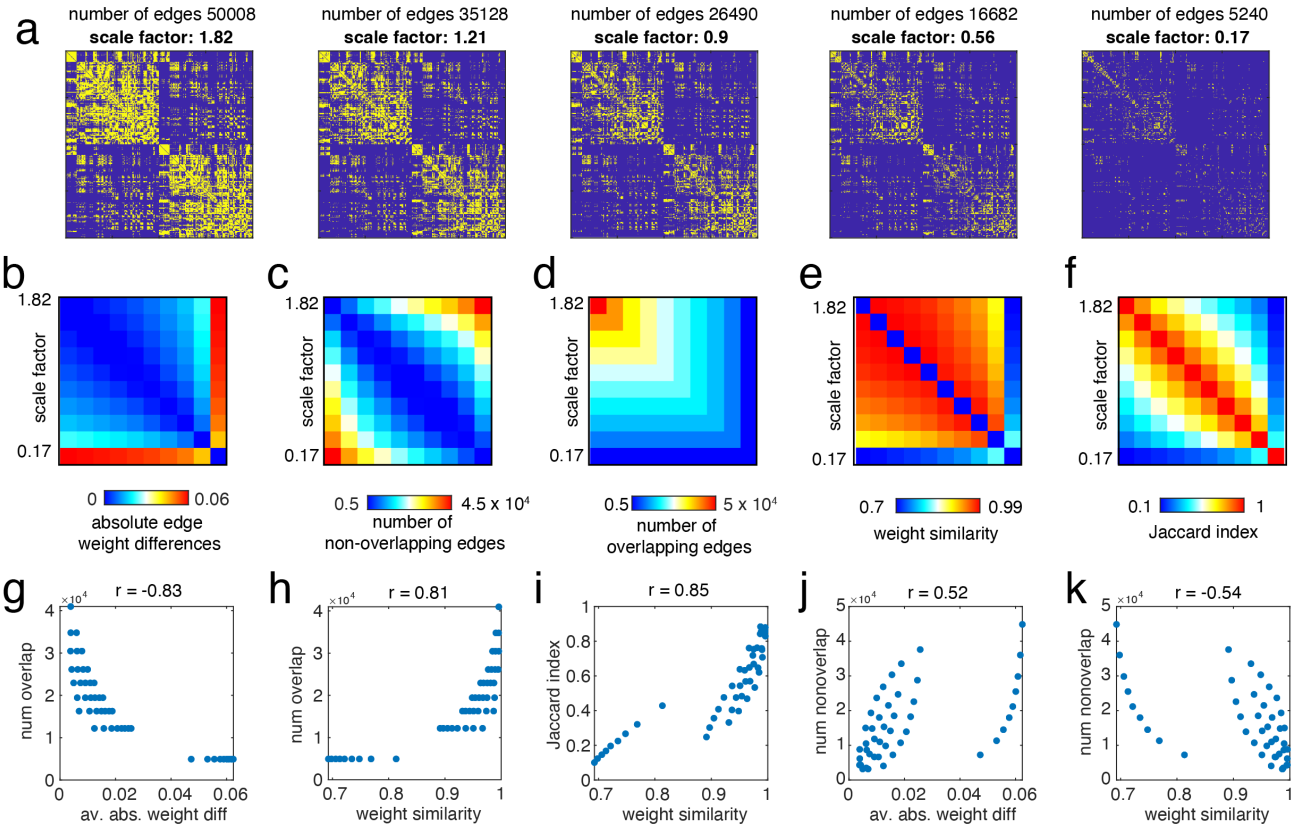

FIG. S19. **Variation in distance-dependent group SC calculation: similar networks produce similar weights.** (*a*) Group structural connectivity matrices produced using a distance-dependent group consensus method described in [8], but varying a scale factor. These scale factors determine the proportion of edges in different distance bins to match with the average subject. We ran our model on the structural matrices produced across ten different scaling factors (1.82, 1.45, 1.21, 1.05, 0.9, 0.79, 0.67, 0.56, 0.4, 0.17) to approximately match the densities in our previous consistency-based analysis. Its important to note the a scale factor of 1 produces the exact same model as the model used in the main text. In the following analyses, we compare the asymmetric, and signed weights of our model across these structural connectomes. More specifically, we compare the weights of edges that are present across all thresholds. (*b*) Matrix of the absolute edge weight differences between each of these scaled models. (*c*) Matrix of the number of non-overlapping edges between each of these scaled SC matrices. (*d*) Matrix of the number of overlapping edges between each of these scaled SC matrices. (*e*) Matrix of the Pearson correlation of weights between each of these scaled models. (*f*) Matrix of the Jaccard similarity between each of these scaled SC matrices. (*g*) Plot showing the negative relationship between the average absolute weight difference of edges and the number of overlapping edges in the scaled SC. (*h*) Plot showing the positive relationship between the weight similarity of edges and the number of overlapping edges in the scaled SC. (*i*) Plot showing the positive relationship between the weight similarity of edges and the Jaccard similarity of the scaled SC. (*j*) Plot showing the positive relationship between the average absolute weight difference of edges and the number of overlapping edges in the scaled SC. (*k*) Plot showing the negative relationship between the weight similarity of edges and the number of non-overlapping edges in the scaled SC.

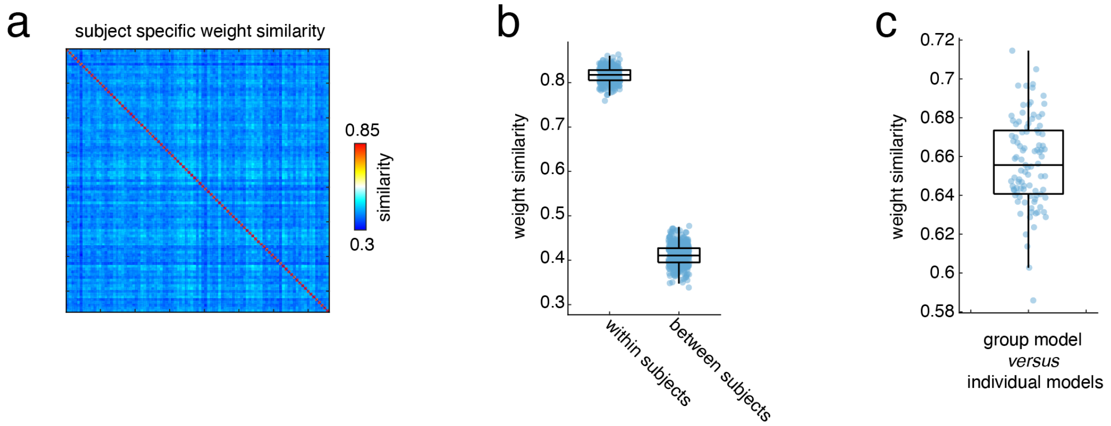

FIG. S20. **Subject-specific model weights are more similar within subjects, but also highly similar to group model weights.** (a) Similarity matrix showing the similarity of all subject-specific models. Subject models were trained in the following manner. Four models were trained per subject. The first model used fMRI scan sessions 2,3 & 4. The second model used fMRI scan sessions 1,3 & 4. Following the general pattern of using three out of four of the scans. Along the block diagonal of this similarity matrix you can see high similarity values that correspond to high within-subject similarity. (b) Box plots showing the within-subject similarity versus the between-subject weight similarity values from the previous similarity matrix. Within subjects are significantly greater than between subject similarity values (two-sample t-test; $p < 10^{-15}$, within $N = 570$, between $N = 71630$). Additionally, between subject similarity values were significantly greater than zero (one-sample t-test; $p < 10^{-15}$). Importantly, the structural connectivity matrices of these between subject models were individualized to each subject, so the weight similarity values correspond to the similarity of the overlapping edges in all subjects structural connectivity matrices. (c) Finally, we trained 95 new subject-specific models (all four fMRI scan sessions for each subjects model), and then compared the weights of edges that existed in all subject specific models with the weights of those edges in the group estimated model weights that was used in the main text (total number of overlapping connections was 9132). We found that these weights were highly similar ($N = 95$).

SUPPLEMENTARY REFERENCES

[1] Roberto F Galán, "On how network architecture determines the dominant patterns of spontaneous neural activity," PloS one **3**, e2148 (2008).

[2] Peng Wang, Ru Kong, Xiaolu Kong, Raphaël Liégeois, Csaba Orban, Gustavo Deco, Martijn P Van Den Heuvel, and BT Thomas Yeo, "Inversion of a large-scale circuit model reveals a cortical hierarchy in the dynamic resting human brain," Science advances **5**, eaat7854 (2019).

[3] Lucas GS Jeub, Olaf Sporns, and Santo Fortunato, "Multiresolution consensus clustering in networks," Scientific reports **8**, 1–16 (2018).

[4] Andrea Lancichinetti and Santo Fortunato, "Consensus clustering in complex networks," Scientific reports **2**, 1–7 (2012).

[5] Mikail Rubinov and Olaf Sporns, "Weight-conserving characterization of complex functional brain networks," Neuroimage **56**, 2068–2079 (2011).

[6] Danielle S Bassett, Eli T Owens, Mason A Porter, M Lisa Manning, and Karen E Daniels, "Extraction of force-chain network architecture in granular materials using community detection," Soft Matter **11**, 2731–2744 (2015).

[7] Justine Y Hansen, Golia Shafiei, Ross D Markello, Kelly Smart, Sylvia ML Cox, Yanjun Wu, Jean-Dominique Gallezot, Étienne Aumont, Stijn Servaes, Stephanie G Scala, *et al.*, "Mapping neurotransmitter systems to the structural and functional organization of the human neocortex," Biorxiv (2021).

[8] Richard F Betzel, Alessandra Griffa, Patric Hagmann, and Bratislav Mišić, "Distance-dependent consensus thresholds for generating group-representative structural brain networks," Network neuroscience **3**, 475–496 (2019).
