# Supplementary material for: A multi-modal, asymmetric, weighted, and signed description of anatomical connectivity
Source: Nat Commun. 2024 Jul 12;15:5865. doi: 10.1038/s41467-024-50248-6 (PMC11245624; doi:10.1038/s41467-024-50248-6)
Supplement: Supplementary file 3 — Reporting Summary [file 41467_2024_50248_MOESM3_ESM.pdf]

nature portfolio

Corresponding author(s): Richard Betzel

Last updated by author(s): Jun 14, 2024

Reporting Summary

Nature Portfolio wishes to improve the reproducibility of the work that we publish. This form provides structure for consistency and transparency in reporting. For further information on Nature Portfolio policies, see our Editorial Policies and the Editorial Policy Checklist.

Statistics

For all statistical analyses, confirm that the following items are present in the figure legend, table legend, main text, or Methods section.

n/a	Confirmed	
☐	☒	The exact sample size (n) for each experimental group/condition, given as a discrete number and unit of measurement
☐	☒	A statement on whether measurements were taken from distinct samples or whether the same sample was measured repeatedly
☐	☒	The statistical test(s) used AND whether they are one- or two-sided<br>*Only common tests should be described solely by name; describe more complex techniques in the Methods section.*
☐	☒	A description of all covariates tested
☐	☒	A description of any assumptions or corrections, such as tests of normality and adjustment for multiple comparisons
☐	☒	A full description of the statistical parameters including central tendency (e.g. means) or other basic estimates (e.g. regression coefficient) AND variation (e.g. standard deviation) or associated estimates of uncertainty (e.g. confidence intervals)
☐	☒	For null hypothesis testing, the test statistic (e.g. F, t, r) with confidence intervals, effect sizes, degrees of freedom and P value noted<br>*Give P values as exact values whenever suitable.*
☒	☐	For Bayesian analysis, information on the choice of priors and Markov chain Monte Carlo settings
☒	☐	For hierarchical and complex designs, identification of the appropriate level for tests and full reporting of outcomes
☐	☒	Estimates of effect sizes (e.g. Cohen's d, Pearson's r), indicating how they were calculated

Our web collection on statistics for biologists contains articles on many of the points above.

Software and code

Policy information about availability of computer code

Data collection	The HCP and NKI data used in this study are publicly available. The authors of this study did not write custom code to collect this data; the authors did not use software for collecting the HCP or NKI data.
Data analysis	Code to produce an asymmetric, weighted and signed connectome with functional and structural data, and to replicate many of our results can be found at: https://github.com/JacobColbyTanner/asymmetric_weighted_and_signed_connectome-main.git

For manuscripts utilizing custom algorithms or software that are central to the research but not yet described in published literature, software must be made available to editors and reviewers. We strongly encourage code deposition in a community repository (e.g. GitHub). See the Nature Portfolio guidelines for submitting code & software for further information.

Data

Policy information about availability of data

All manuscripts must include a data availability statement. This statement should provide the following information, where applicable:

- Accession codes, unique identifiers, or web links for publicly available datasets
- A description of any restrictions on data availability
- For clinical datasets or third party data, please ensure that the statement adheres to our policy

All of the human neuroimaging datasets used here are publicly available. Data from both of the Human Connectome Project datasets can be downloaded at:

(https://db.humanconnectome.org/). Information on accessing the NKI dataset can be found at: (http://fcon_1000.projects.nitrc.org/indi/enhanced/neurodata.html). All data generated and used in the main figures of this study are provided in the Supplementary Information/Source Data file.

Research involving human participants, their data, or biological material

Policy information about studies with human participants or human data. See also policy information about sex, gender (identity/presentation), and sexual orientation and race, ethnicity and racism.

Reporting on sex and gender	We do not expect the results of the presented brain mapping analyses to have sex nor gender effects, and we did not perform analyses where either of these variables served as the dependent variable. The analyses presented collapse over these variables.
Reporting on race, ethnicity, or other socially relevant groupings	We did not explore race, ethnicity, or other socially relevant groupings in this study.
Population characteristics	The Human Connectome Project (HCP) aimed to collect healthy adult twins, ages 22-25 years old (Van Essen, 2012). The definition of "healthy" was broad, in order to collect a sample representative of the United States population in terms of behavior, ethnic, and socioeconomic diversity. In this study, a provided subset of subjects that were scanned at the Center for Magnetic Resonance Research at the University of Minnesota were used. The NKI is a prospective data collection project (initialized March 2012) aiming to collect a large scale (number of subjects> 1000) community-ascertained lifespan sample, within Rockland County, New York. The study coordinators aimed to collect a sample with representative age, ethnicity, and socioeconomic status of Rockland County. Further details about population characteristics can be found in Nooner et al. (2012).
Recruitment	HCP subjects were recruited from the Missouri Department of Health and Senior Services Bureau of Vital Records. NKI subjects were recruited based on zip code (e.g. advertisement flyer mailings, posting of recruitment materials in local shops and meeting places). As the HCP is being used as a publicly available source of data that the manuscript authors did not collect, there is no risk for self-section biases or other biases associated with the data collection.
Ethics oversight	The HCP cohort data collection was approved by the Washington University Institutional Review Board. The NKI cohort data was approved by Nathan Klein Institute Institutional Review Board and the Montclair State University Institutional Review Board.

Note that full information on the approval of the study protocol must also be provided in the manuscript.

Field-specific reporting

Please select the one below that is the best fit for your research. If you are not sure, read the appropriate sections before making your selection.

☒ Life sciences ☐ Behavioural & social sciences ☐ Ecological, evolutionary & environmental sciences

For a reference copy of the document with all sections, see nature.com/documents/nr-reporting-summary-flat.pdf

Life sciences study design

All studies must disclose on these points even when the disclosure is negative.

Sample size	For the HCP cohort, the sample size was determined by the number of available subjects collected by the HCP investigators. No statistical methods were used to predetermine sample sizes, but our sample sizes are similar to those reported in previous publications and represent all usable and complete data at the time of download, excluding subjects based on quality control criteria. We used 95 subjects from the 100 unrelated subject cohort. 5 subjects were excluded based on data quality.<br><br>For the NKI cohort, we considered data from all subjects (N = 542) and we then used motion filtering to determine which subjects had high-quality and low-motion data. This left us with a total of 474 subjects.
Data exclusions	For neuroimaging data, data exclusions were based on data quality, to filter out scans with excessive motion or image artifact, and based on data completeness, to ensure that each subject that the appropriate meta-data, and functional scans. For HCP, our exclusion criteria was as follows: where each spike is defined as relative framewise displacement of at least 0.25 mm, we excluded subjects who fulfill at least 1 of the following criteria: greater than 15% of time points spike, average framewise displacement greater than 0.2 mm; contains any spikes larger than 5mm. or NKI, subjects were considered for data exclusion based on having a complete set ofTlw, resting state, diffusion images, and meta-data, as well as the quality of the aforementioned images. The ENIGMA QC FreeSurfer tools (http://enigma.ini.usc.edu/protocols/imaging-protocols/), M RIQC (https://m riqc.readthedocs.io/en/stable/), eddy_ qc (https://fsl. fmrib.ox.ac. u k/fsl/fslwi ki/eddyqc/UsersG uide), and QAscripts (https://www.med.upenn.edu/cmroi/qascripts.html) were used to derive image quality metrics to assess data quality of the Tlw, resting state, and diffusion images. Details of exclusion criteria can be found in the manuscript section "Quality Control". Of the 567 subjects who passed image quality control, only 542 subjects had age meta-data available.
Replication	Many of the results of our study were replicated in mice. Additionally, we have generated an extensive toolbox that can be used to easily replicate all of our results on different datasets (see above).

Randomization	Subjects were not partitioned into groups. Data from each cohort (HCP, NKI) were analyzed separately. This choice was made so as to not mix data across MRI machine and MRI acquisition parameters.
Blinding	Data analysis was not performed blind to the conditions of the experiments. Blinding was not relevant because subjects were not evaluated based on group membership and blinding was not applicable to the whole-group analyses reported in this study.

Reporting for specific materials, systems and methods

We require information from authors about some types of materials, experimental systems and methods used in many studies. Here, indicate whether each material, system or method listed is relevant to your study. If you are not sure if a list item applies to your research, read the appropriate section before selecting a response.

Materials & experimental systems

n/a	Involved in the study	
☒	☐	Antibodies
☒	☐	Eukaryotic cell lines
☒	☐	Palaeontology and archaeology
☐	☒	Animals and other organisms
☒	☐	Clinical data
☒	☐	Dual use research of concern
☒	☐	Plants

Methods

n/a	Involved in the study	
☒	☐	ChIP-seq
☒	☐	Flow cytometry
☐	☒	MRI-based neuroimaging

Animals and other research organisms

Policy information about studies involving animals; ARRIVE guidelines recommended for reporting animal research, and Sex and Gender in Research

Laboratory animals	The rsfMRI dataset used in this<br>work consists of n = 19 scans in adult male CS 7BL/6J mice
Wild animals	This study did not involve wild animals.
Reporting on sex	Only male mice were used in this study.
Field-collected samples	This study did not involve samples collected from the field.
Ethics oversight	All in vivo experiments were conducted in accordance<br>with the Italian law (DL 2006/2014, EU 63 /2010, Ministero della Sanit'a, Roma) and the recommendations in the Guide for the Care and Use of Laboratory Animals of<br>the National Institutes of Health. Animal research protocols were reviewed and consented by the animal care committee of the Italian Institute ofTechnology and Italian Ministry of Health.

Note that full information on the approval of the study protocol must also be provided in the manuscript.

Plants

Seed stocks	Not applicable.
Novel plant genotypes	Not applicable.
Authentication	Not applicable.

Magnetic resonance imaging

Experimental design

Design type	In this paper, we analyzed four separate MRI datasets: Human Connectome Project 3T data (HCP3T), Human Connectome Project 7T data (HCP7T), Nathan Kline Institute data (NKI), and mouse MRI data. The HCP3T, NKI and

mouse data that we analyzed were resting-state scans. The HCP3T, HCP7T, and NKI also include diffusion weighted scans. The 7T data that we analyzed included both resting state and movie-watching scans of the same set of subjects.

Design specifications

This is described below in the "Sequence & imaging parameters" section.

Behavioral performance measures

Behavioral performance data were not taken into consideration in this study.

Acquisition

Imaging type(s)

Functional, diffusion

Field strength

HCP: 3T, 7T (which used in each analysis is specified in the paper), NKI: 3T, Mouse MSI: 7T

Sequence & imaging parameters

HCP 7T: Collected on a 7T Siemens Magnetom scanner with a 32-channel head coil. All 7T fMRI data was acquired with a gradient-echo planar imaging sequence (TR = 1000 ms, TE = 22.2 ms, flip angle = 45◦ , 1.6 mm isotropic voxel resolution, multi-band factor = 5, image acceleration factor = 2, partial Fourier sample = 7/8, echo spacing = 0.64 ms, bandwidth = 1924 Hz/Px). Four resting state data runs were collected, each lasting 15 minutes (frames = 900), with eyes open and instructions to fixate on a cross. Four movie watching data runs were collected, each lasting approximately 15 minutes (frames = 921, 918, 915, 901), with subjects passively viewing visual and audio presentations of movie scenes. Movies consisted of both freely available independent films covered by Creative Commons licensing and Hollywood movies prepared for analysis [114]. For both resting state and movie watching data, two runs were acquired with posterior-to-anterior phase encoding direction and two runs were acquired with anterior-to-posterior phase encoding direction.

HCP3T: Images were collected on a 3T Siemens Connectome Skyra with a 32-channel head coil. Subjects underwent two T1-weighted structural scans, which were averaged for each subject (TR = 2400 ms, TE = 2.14 ms, flip angle = 8◦, 0.7 mm isotropic voxel resolution). Subjects underwent four resting state fMRI scans over a two-day span. The fMRI data was acquired with a gradient-echo planar imaging sequence (TR = 720 ms, TE = 33.1 ms, flip angle = 52◦, 2 mm isotropic voxel resolution, multiband factor = 8). Each resting state run duration was 14:33 min, with eyes open and instructions to fixate on a cross. Finally, subjects underwent two diffusion MRI scans, which were acquired with a spin-echo planar imaging sequence (TR = 5520 ms, TE = 89.5 ms, flip angle = 78◦ , 1.25 mm isotropic voxel resolution, b-vales = 1000, 2000, 3000 s/mm2 , 90 diffusion weighed volumes for each shell, 18 b = 0 volumes). These two scans were taken with opposite phase encoding directions and averaged.

NKI: The fMRI data was acquired with a gradient-echo planar imaging sequence (TR = 645 ms, TE = 30 ms, flip angle = 60◦ , 3 mm isotropic voxel resolution, multiband factor = 4). This resting state run lasted approximately 9:41 seconds, with eyes open and instructions to fixate on a cross. Subjects underwent one diffusion MRI scan (TR = 2400 ms, TE = 85 ms, flip angle = 90◦ , 2 mm isotropic voxel resolution, 128 diffusion weighted volumes, b-value = 1500 s/mm2 , 9 b = 0 volumes).

Mouse MRI: Briefly, rsfMRI data were acquired on a 7.0-T scanner (Bruker BioSpin, Ettlingen) equipped with BGA-9 gradient set, using a 72-mm birdcage transmit coil, and a four-channel solenoid coil for signal reception. Single-shot BOLD echo planar imaging time series were acquired using an echo planar imaging sequence with the following parameters: repetition time/echo time, 1000/15 ms; flip angle, 30◦ ; matrix, 100×100; field of view, 2 × 2 cm2; 18 coronal slices; slice thickness, 0.50 mm; 500 (n = 21) or 1500 (n = 19) volumes; and a total rsfMRI acquisition time of 30 min

Area of acquisition

All scans were of the whole brain.

Diffusion MRI ☒ Used ☐ Not used

Parameters

HCP: 3 shells (bvals: 1000, 2000, 3000), 90 directions per shell, 18 unweighted volumes; NKI: 1 shell (bval: 1500), 128 directions, 9 unweighted volumes

Preprocessing

Preprocessing software

HCP3T: Briefly, T1w images were aligned to MNI space before undergoing FreeSurfer's (version 5.3) cortical reconstruction workflow, as part of the HCP Pipeline's PreFreeSurfer, FreeSurfer, and PostFreeSurfer steps. Functional images were corrected for gradient distortion, susceptibility distortion, and motion, and then aligned to the corresponding T1w with one spline interpolation step. This volume was further corrected for intensity bias and normalized to a mean of 10000. This volume was then projected to the 2mm 32k fs LR mesh, excluding outliers, and aligned to a common space using a multi-modal surface registration [106]. The resultant cifti file for each HCP subject used in this study followed the file naming pattern:* Atlas MSMAll hp2000 clean.dtseries.nii. These steps are performed as part of the HCP Pipeline's fMRIVolume and fMRISurface steps. Each minimally preprocessed fMRI was linearly detrended, band-pass filtered (0.008-0.008 Hz), confound regressed and standardized using Nilearn's signal.clean function, which removes confounds orthogonally to the temporal filters. The confound regression strategy included six motion estimates, mean signal from a white matter, cerebrospinal fluid, and whole brain mask, derivatives of these previous nine regressors, and squares of these 18 terms. Spike regressors were not applied. Following these preprocessing operations, the mean signal was taken at each time frame for each node, as defined by the Schaefer 200 parcellation [48] in 32k fs LR space. Diffusion images were normalized to the mean b0 image, corrected for EPI, eddy current, and gradient non-linearity distortions, and motion, and aligned to subject anatomical space using a boundary-based registration as part of the HCP pipeline's Diffusion Preprocessing step. In addition to HCP's minimal preprocessing, diffusion images were corrected for intensity non-uniformity with N4BiasFieldCorrection [107]. The Dipy

toolbox (version 1.1) [108] was used to fit a multi-shell multi-tissue constrained spherical deconvolution [109] to the data with a spherical harmonics order of 8, using tissue maps estimated with FSL's fast [110]. Tractography was performed using Dipy's Local Tracking module [108]. Multiple instances of probabilistic tractography were run per subject [111], varying the step size and maximum turning angle of the algorithm. Tractography was run at step sizes of 0.25 mm, 0.4 mm, 0.5 mm, 0.6 mm, and0.75 mm with the maximum turning angle set to 20◦
.
Additionally, tractography was run at maximum turning angles of 10◦, 16◦, 24◦, and 30◦ with the step size set to 0.5 mm. For each instance of tractography, streamlines were randomly seeded three times within each voxel of a white matter mask, retained if longer than 10 mm and with valid endpoints, following Dipy's implementation of anatomically constrained tractography [112], and errant streamlines were filtered based on the cluster confidence index [113]. For each tractography instance, streamline count between regions-of-interest were normalized by dividing the count between regions by the geometric average volume of the regions. Since tractography was run nine times per subject, edge values were collapsed across runs. To do this, the weighted mean was taken with weights based on the proportion of total streamlines at that edge. This operation biases edge weights towards larger values, which reflect tractography instances better parameterized to estimate the geometry of each connection.

HCP7T: Briefly, T1w images were aligned to MNI space before undergoing FreeSurfer's (version 5.3) cortical reconstruction workflow, as part of the HCP Pipeline's PreFreeSurfer, FreeSurfer, and PostFreeSurfer steps. 7T fMRI images were downloaded after correction and reprocessing announced by the HCP consortium in April, 2018. fMRI images were corrected for gradient distortion, susceptibility distortion, and motion, and then aligned to the corresponding T1w with one spline interpolation step. This volume was further corrected for intensity bias and normalized to a mean of 10000. This volume was then projected to the 2mm 32k fs LR mesh, excluding outliers, and aligned to a common space using a multi-modal surface registration [106]. The resultant cifti file for each HCP subject used in this study followed the file naming pattern: * Atlas MSMAll hp2000 clean.dtseries.nii. These steps are performed as part of the HCP Pipeline's fMRIVolume and fMRISurface steps. Resting state and moving watching fMRI images were nuisance regressed in the same manner. Each minimally preprocessed fMRI was linearly detrended, band-pass filtered (0.008-0.25 Hz), confound regressed and standardized using Nilearn's signal.clean function, which removes confounds orthogonally to the temporal filters. The confound regression strategy included six motion estimates, mean signal from a white matter, cerebrospinal fluid, and whole brain mask, derivatives of these previous nine regressors, and squares of these 18 terms. Spike regressors were not applied. Following these preprocessing operations, the mean signal was taken at each time frame for each node, as defined by the Schaefer 400 parcellation [48] in 32k fs LR space.

NKI: TT1-weighted images were submitted to FreeSurfer's cortical reconstruction workflow (version 6.0). The FreeSurfer results were used to skull strip the T1w, which was subsequently aligned to MNI space with 6 degrees of freedom. fMRI preprocessing was performed using the fMRIPrep version 1.1.8 [118]. The following description of fMRI preprocessing is based on fMRIPrep's documentation. This workflow utilizes ANTs (2.1.0), FSL (5.0.9), AFNI (16.2.07), FreeSurfer (6.0.1), nipype [119], and nilearn [120]. Each T1w was corrected using N4BiasFieldCorrection [107] and skull-stripped using antsBrainExtraction.sh (using the OASIS template). The ANTs derived brain mask was refined with a custom variation of the method to reconcile ANTs-derived and FreeSurfer-derived segmentations of the cortical graymatter of Mindboggle [121]. Brain tissue segmentation of cerebrospinal fluid (CSF), white-matter (WM) and gray-matter (GM) was performed on the brain-extracted T1w using fast [110]. Functional data was slice time corrected using 3dTshift from AFNI and motion corrected using FSL's mcflirt. "Fieldmap-less" distortion correction was performed by co-registering the functional image to the same-subject T1w with intensity inverted [122] constrained with an average fieldmap template [123], implemented with antsRegistration. This was followed by co-registration to the corresponding T1w using boundary-based registration [124] with 9 degrees of freedom, using bbregister. Motion correcting transformations, field distortion correcting warp, and BOLD-toT1w transformation warp were concatenated and applied in a single step using antsApplyTransforms using Lanczos interpolation. Frame-wise displacement [125] was calculated for each functional run using the implementation of Nipype. The first four frames of the BOLD data in the T1w space were discarded. Diffusion images were preprocessed following the "DESIGNER" pipeline using MRTrix (3.0) [126, 127], which includes denoising, Gibbs ringing and Rician bias correction, distortion and eddy current correction [128] and B1 field correction. DWI were then aligned to their corresponding T1w and the MNI space in one interpolation step with B-vectors rotated accordingly. Local models of white matter orientation were estimated in a recursive manner [129] using constrained spherical deconvolution [109] with a spherical harmonics order of 8. Tractography was performed using Dipy's Local Tracking module [108]. Probabilistic streamline tractography was seeded five times in each white matter voxel. Streamlines were propagated with a 0.5 mm step size and a maximum turning angle set to 20◦. Streamlines were retained if longer than 10 mm and with valid endpoints, following Dipy's implementation of anatomically constrained tractography [112]. Streamline count between regions-of-interest were normalized by dividing the count between regions by the geometric average volume of the regions.

Normalization

See above.

Normalization template

For HCP, fMRI data was analyzed after linear alignment (AC-PC) to the FSL MNI template. For NKI, fMRI data was analyzed in each subject's T1w space.

Noise and artifact removal

See "Processing software" section

Volume censoring

See "Processing software" section

Statistical modeling & inference

Model type and settings: n/a

Effect(s) tested: n/a

Specify type of analysis: ☒ Whole brain ☐ ROI-based ☐ Both

Statistic type for inference (See Eklund et al. 2016): n/a

Correction: n/a

Models & analysis

n/a	Involved in the study
☐	☒ Functional and/or effective connectivity
☐	☒ Graph analysis
☐	☒ Multivariate modeling or predictive analysis

Functional and/or effective connectivity: This paper is focused on the development and exploration of a new model for connectome weighting that sits in-between functional and structural networks. As such, there is some relationship between this new connectome and both functional and structural connectivity networks.

Graph analysis: We used a number of methods for graph analysis, including but not limited to: shortest path analysis, and community detection (e.g., modularity maximization). Such tools can be found in the openly available Brain Connectivity Toolbox.

Multivariate modeling and predictive analysis: Our newly weighted connectome is weighted using a constrained multiple linear regression model where the activity of any given node is predicted by the past activity of its structurally connected neighbors.
